# Health Status and the Demand for Healthcare among the Elderly in the Rural Quoc-Oai District of Hanoi in Vietnam

**DOI:** 10.1155/2017/4830968

**Published:** 2017-09-24

**Authors:** Kyung-Sook Bang, Sunghee H. Tak, Juhwan Oh, Jinseon Yi, Soo-Young Yu, Truong Quang Trung

**Affiliations:** ^1^The Research Institute of Nursing Science, College of Nursing, Seoul National University, Seoul, Republic of Korea; ^2^JW Lee Center for Global Medicine, College of Medicine, Seoul National University, Seoul, Republic of Korea; ^3^College of Nursing, Seoul National University, Seoul, Republic of Korea; ^4^Faculty of Nursing and Midwifery, Hanoi Medical University, Hanoi, Vietnam

## Abstract

**Background:**

Vietnam is experiencing an unprecedented demographic transition. Its proportion of elderly people is growing rapidly.

**Objective:**

This study explored the health status and health-related quality of life (HRQoL) of rural elderly Vietnamese and assessed their needs for healthcare services.

**Design:**

This study used a survey with stratified proportion sampling and quota assignment. In 2016, data was collected from 713 people in the rural Quoc-Oai district of Hanoi aged 60 or older.

**Results:**

The mean age of the respondents was 70.9. Both self-rated health status and functional status decreased with age. Women reported more functional limitations than men. Musculoskeletal disorders were the most frequently reported chronic diseases, followed by hypertension, gastrointestinal diseases, and cardiovascular diseases. Age, self-rated health status, BMIs, and the number of noncommunicable diseases (NCDs) were found to be significant determinants of HRQoL, after controlling for socioeconomic effects. More than half the respondents requested more healthcare information, particularly on disease management.

**Conclusions:**

Vietnam's healthcare system is being challenged to make health services easily accessible and meet the growing needs for chronic illness management, risk reduction, promoting healthy lifestyles, and improving the aging population's quality of life.

## 1. Introduction

The world's population is aging, and this trend is also evident in Vietnam, where the proportion of people over 60 years of age has increased rapidly in recent decades, from 6.7% in 1979 to 10% in 2013. Further influencing this trend is confirmation that life expectancy at birth increased from 66 years in 1990 to 76 years by 2012 [1]. Meanwhile, epidemiological evidence has shown that even in developing countries the most prevalent diseases are no longer communicable diseases, but rather noncommunicable diseases (NCDs). The World Health Organization's 2014 NCD Country Profile for Vietnam estimated that NCDs account for 73% of all deaths [2]. Mean systolic blood pressure readings have been increasing since 1980, and mean total cholesterol levels have also been increasing since 1996. Stroke and ischemic heart disease were the leading causes of death in 2012 [2].

These data highlight elderly people's need to adopt lifestyle modifications for their health. However, lifestyle changes are not made easily. Systematic education, motivation, and continuous monitoring by health professionals might be very helpful for encouraging lifestyle changes. In addition, functional status and health status are both highly related to the quality of life, and especially so for the elderly [3]. In one study, almost ten percent of the respondents reported a need for some help conducting activities of daily living (ADL), while over two-thirds of the elderly in rural Vietnam indicated that they needed instrumental or intellectual ADL support [4].

With rapid industrialization, young people moved to urban areas for employment, and, over time, the family structure in rural areas has changed significantly. In 1998, the proportion of families comprised only of elderly spouses was 12.7%, and the proportion of elderly people aged 60 and over living alone was 4.93%. However, these proportions increased to 21.47% and 6.14%, respectively, in 2008 [5]. The combined percentage of elderly people who live on their own as couples and those who live alone continues to increase over time. In 2004 and 2005, L. Nguyen and T. H. Nguyen (2010) analyzed the secondary data of 979 elderly participants from five provinces in Vietnam (Quang Nam, Ho chi minh City, Thanh Hoa, Son La, and Kon Tum). Among 938 responders who chose home-based care when suffering from illness, 74.2% (696) of participants received care from their children, 22% (206) provided self-care, and 2.1% (20) received care from friends or others. Only 0.5% (5) of the participants hired a housemaid to take care for them. These numbers may increase or differ from the current situation, because of economic development, industrialization, and urbanization within Vietnam [6].

While elderly people in urban areas can benefit directly from facilities for the elderly [7], more than 70% of Vietnamese seniors live in rural areas, where limited financial resources are available [8]. The elderly who lived in rural areas were earlier cared for by their families; however, they can no longer rely solely on their families, and other sources of support should be proposed. The model of caring for elderly people within the community differed across different geographical regions within Vietnam [9]. According to one report, every elderly person suffers 2.69 diseases on average, and most of the elderly living in rural areas have poorer health status than their counterparts in urban areas [10]. It is evident that better planning is required to provide the aging populations in rural communities with the healthcare services they need.

To date, studies on the health status of elderly populations in Vietnam's rural areas have been very limited. Conducting these studies requires that the targeted population's functional status (ADL) and health-related quality of life (HRQoL) be evaluated and the factors that influence these measures be identified. Furthermore, their needs for healthcare services should be assessed, and strategies and policies for improving their health should be developed.

This study of elderly people in Vietnam's rural areas has two main objectives. The first is to explore this population's health status and HRQoL and to confirm the significant determinants. The second objective is to assess their needs for healthcare services.

## 2. Methods

### 2.1. Study Setting

This study was conducted in 2016 as part of a joint cohort study for a Health System Strengthening project led by collaborators from two universities in Vietnam and one in Korea. The project team selected the Quoc-Oai district of Hanoi—located 30 km from Western Hanoi—as the research area and conducted a community-based survey to generate baseline data. The area is divided into two strata, namely, lowland and mountainous areas. All clusters and corresponding population numbers were listed using a probability-proportional-to-size (PPS) technique [11] that is a widely used and recommended method for obtaining a representative national sample. A stratified multistage cluster sampling technique was used to identify households for this study. Based on a three-stage cluster sampling strategy, 2,400 households were randomly selected from 30 clusters (villages), which in turn had been randomly selected from 2 strata: lowlands and mountains. One person aged between 15 and 60 years was randomly selected from each household, and if there was more than one person over 60 living in a household, another person aged over 60 years was randomly sampled.

### 2.2. Sampling and Sample Size

The sample size selected for this survey was based on the formula suggested by the WHO [12], and a proportion of each population and the national figures estimated by Ho chi minh Medical University/WHO research. Based on this calculation, a sample size of approximately 1,100 people was required. In most prevalence surveys, a design effect of 1.5~2 is reasonable [13], so in this study the design effect of 2 was applied. Based on an expected nonresponse rate of about 10%, the final sample size was estimated to be 2,400 households.

**Figure 1:**
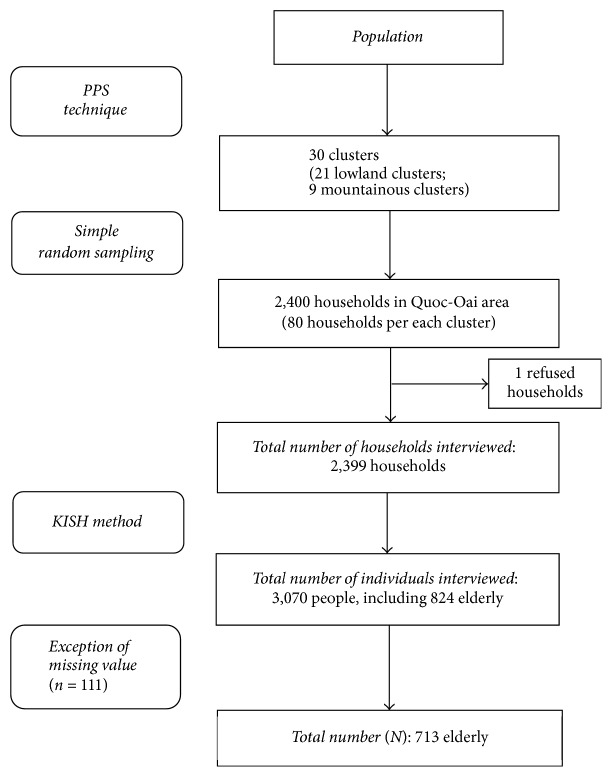
Sampling and data collection process.

The number of households that responded to the survey was 2,399, and the response rate was 99.9%. Individual interviewees were chosen using the Kish Method [14], and, in each selected household, the data collector interviewed the head of the household using the household questionnaire, and two household members using the questionnaire designed for individuals (one individual between 15 and 59 years of age, and one individual 60 years of age or older). The total number of individuals who responded to the survey was 3,070, and 824 individuals who were 60 years of age or older were selected to be study participants. Responses with incomplete data were removed, and the remaining 713 respondents were included in the final analysis. The sampling procedure is illustrated in Figure 1.

### 2.3. Data Collection

Face-to-face interviews were conducted with participants using structured questionnaires. The questionnaires were divided into sections for households and individuals. After the survey had been completed, the data collected from the two surveys were consolidated. Double data-entry using Epidata 3.1 was performed, to evaluate inconsistent values for any variables. To ensure the study's accuracy, any responses with missing or irrelevant values were deleted.

### 2.4. Variable Measurements and Instruments

We used age, gender, ethnicity, location, marital status, educational level, religion, working status, and household size as demographic variables. Self-rated health status was measured using a 5-point Likert scale, which was reclassified into three categories: good, normal, and bad. BMIs were calculated using self-reported heights and weights and categorized on the basis of Asian-specific BMI cut-offs [15]. Chronic disease prevalence was measured with a multiple-choice questionnaire that asked the following question: “In the past 12 months, have you been diagnosed by a doctor with one or more of the following chronic diseases?”

Social capital encompasses different characteristics, such as social networks, social participation, social support, social cohesion, attitudes, and trust [16]. For this study, social capital was measured using an instrument developed in 2012 [17]. It consists of two domains: the first evaluates whether there is a relationship based on general trust or support between neighbors in everyday life, and the second asks whether there is a relationship based on trust in emergency situations. The first domain asks four questions with regard to general situations: they relate to a “willingness to help each other,” the “perceived sense of living in a close-knit village,” the “perceived level of a neighbor's trust,” and “how much neighbors can work together.” Respondents chose from one of five levels to indicate their level of agreement with these statements. The second domain consisted of four questions related to individuals in urgent need of help: they included “the possibility of receiving help from one's neighbor,” “willingness to help,” “the number of people who give sincere advice,” and “the number of people from whom you can borrow money.” The possible responses again consisted of four levels: four was the highest, and one was the lowest. Each score for the second domain was recalculated with a weighted value that changed it to its original score by 1.25.

The participants' functioning assessment was measured using a modified version of a generic assessment instrument for health and disability developed as part of a WHO Disability Assessment Schedule (WHODAS) [18]. This instrument consists of ten items asking questions about five domains: mobility, self-care, getting along, life activities, participation, and cognition. The higher one's score on the functioning assessment, the more limited the individual's physical activity level.

The Vietnamese version of the EQ-5D-3L questionnaire [19] was used to measure the health-related quality of life (HRQoL) of the elderly in rural areas. This questionnaire is comprised five dimensions, namely, mobility, self-care, usual activities, pain/discomfort, and anxiety/depression. Responses permitted for each dimension had three levels: no problems, some problems, and extreme problems. Even though the Vietnamese version of EQ-5D-3L was used, because it lacks the preference value set developed for Vietnam, the time trade-off valuation set from South Korea [15] was used in this study. The EQ-5D-3L index ranged from −0.171 to 1, and higher values indicated a better health-related quality of life.

Participants' needs for health information and home healthcare services were confirmed during interviews conducted using open-ended questions. Participants who responded by saying that they needed information with regard to their health status described what types of health information they wanted to receive. If they had any needs for home healthcare services—including rehabilitation—participants were asked to describe the health problems for which would they like to receive services. Interviewers summarized participants' answers and coded the responses using short words.

### 2.5. Data Analysis

SPSS Statistics 23 software was used for the statistical analysis. Descriptive statistical analyses were used to describe the socioeconomic characteristics, health status, prevalence of NCDs, social capital, functioning assessment, and health-related quality of life. Student's *t*-test and a one-way ANOVA were used to compare the differences between these characteristics in a functioning assessment and the HRQoL. For the post hoc test, Fisher's least significant difference (LSD) was applied. Multiple regression analysis was performed to detect the effects of factors on the HRQoL index. The significance level was set at *p* < 0.05.

The responses to open-ended questions for healthcare needs were analyzed using NVivo 11 software. The responses most frequently in all the subjective texts were selected and a weighted percentage was calculated, after which the authors created domains and categorized the texts into these domains.

### 2.6. Ethical Considerations

The Institutional Review Board of the Hanoi School of Public Health approved this study in 2016. After they had been informed of the purpose of the study and their rights, informed consent was obtained from all participants. They were told that they could refuse to participate, or choose to withdraw from an interview, at any time. Confidentiality was assured, by coding participants' responses.

## 3. Results

### 3.1. Demographic Characteristics

An overview of the distribution of study participants is provided in Table 1. The average age of study participants was 70.9, and the majority of them were between 60 and 69 years of age (52.9%). Of the 713 participants, 58.6% were women. Participants' ethnicity was primarily Kinh (81.3%), which is the largest ethnic group in Vietnam [20], and most of the participants lived in plain areas (77.8%). More than half the participants were married or reunited (58.5%), and the next-largest group was widowed (37.2%). In terms of their education levels, more than one-third had not completed primary school (34.6%), and 28.5% and 25.5%, respectively, had completed primary school and secondary school. Most participants had no religion (98.2%); those who were working among the participants (76.2%) were three times more than those who did not work (23.8%). The proportion of people living in the middle to the poorest households was higher than those in the richer or richest wealth quintiles. The percentage of those with health insurance was 72.5%.

### 3.2. Health Status and Chronic Disease Prevalence

Self-rated health status, body mass indexes (BMIs), and the prevalence of a chronic disease diagnosis by a doctor within 12 months are summarized in Table 2. The mean score of the self-rated health status was 58.4 (it ranged from 2 to 100) and tended to decrease with aging. Participants' average BMI scores were 19.9. Most of the participants (66.9%) were categorized within the “normal range” according to current WHO BMI cut-off points [21], but 16.3% of participants were mildly underweight (17.0–18.49 kg/m^2^), 5.0% were moderately underweight (16.0–16.9 kg/m^2^), and 7.9% of participants were severely underweight (<16 kg/m^2^). The total mean score for social capital in this study was 3.52 (0.487) on a 5-point scale. The average score for the first domain (trust in daily life), which was asking about one's generalized trust in everyday life, was 4.17 (0.490). The other domain, which asked questions concerning how one felt about neighbors' attitudes toward helping them when they were in an emergency situation, scored 3.00 (0.628). There are no statistically significant relationships between social capital and other variables such as age, gender, and wealth quintile. Musculoskeletal disorders were the most frequently reported chronic disease among all age groups (50.8%), followed by hypertension (31.6%), gastrointestinal diseases (17.3%), and cardiovascular diseases (7.9%). Very few cases of cancer or diabetes were reported.

### 3.3. Functional Status

The distribution of participants on the basis of the functional status of different groups is presented in Table 3. Functioning assessment scores were significantly different between gender and age groups, and the number of NCDs was significantly associated with functional status. Participants with more than three NCDs scored lowest in terms of their functioning status.

### 3.4. Health-Related Quality of Life

The mean indices of the health-related quality of life (HRQoL) as measured by EQ-5D-3L are presented in Table 4. The HRQoL decreased significantly with age. Men and Non-Kinh had higher indices than women and Kinh. Married or reunited people had higher HRQoLs than those who were widowed. The group with less than a primary school education had lower indices than other groups. HRQoL indices increased with a higher self-rated health status. Participants within a normal BMI range had higher indices than those who were underweight, and particularly those who were severely underweight had significantly lower indices than those who were moderately underweight, mildly underweight, and within the normal weight range group. A greater number of noncommunicable diseases were associated with lower HRQoL indices.

Multiple regression analyses were performed to identify the determining factors of elderly participants' HRQoL (Table 5). Model 1 used socioeconomic variables as independents, and Model 2 included additional variables to examine the effects of health-related factors after controlling socioeconomic variables. As shown in Table 5, the factors of age, self-rated health status, BMI, and number of NCDs were significant determinants of elderly participants' HRQoL.

### 3.5. Healthcare Needs

#### 3.5.1. Needed Healthcare Related Information

**Figure 2:**
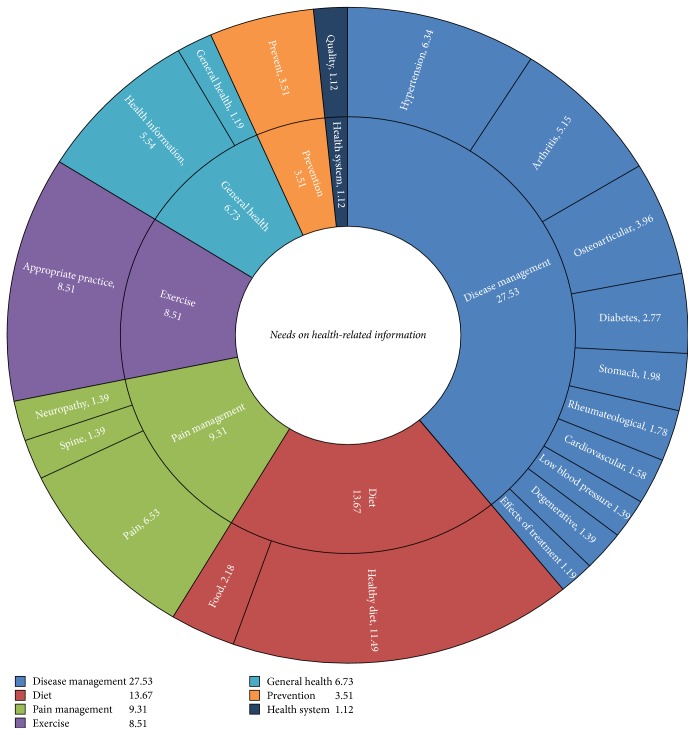
Needs on health-related information.

The number of respondents who expressed a need for health-related information was 387 (54.3%). These needs were categorized into seven areas: disease management, diet, pain management, exercise, general health, disease prevention, the health system, and others (Figure 2). The information requested most often pertained to disease management (27.53%). Information was also requested for chronic diseases such as hypertension, arthritis, musculoskeletal diseases, diabetes, stomach, and rheumatoid diseases. Older adults were most interested in degenerative diseases than other acute diseases. In particular, the category of pain management accounted for 9.3% of the requests. The respondents expressed a need for healthy lifestyle–related information on diet (13.67%) and exercise (8.51%). General health information (6.73%) and that for disease prevention (3.5%) were also desired by the aged. Lastly, requests for practical information to help them assess a health facility's quality accounted for 1.12% of the requested information.

#### 3.5.2. Needed Home Healthcare Services

**Figure 3:**
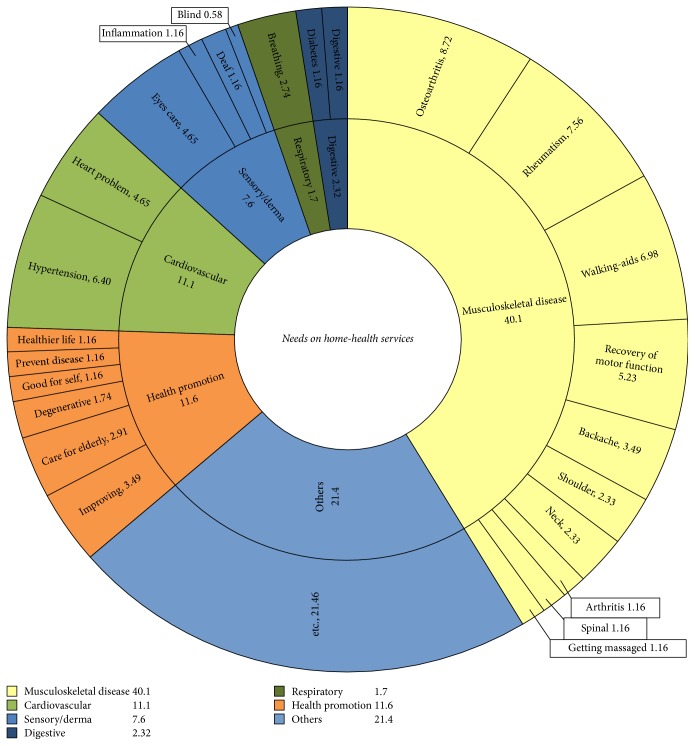
Needs on home health care services.

The number of participants who needed home healthcare services including rehabilitation was 154 (21.6%). The health problems for which respondents want home healthcare services or rehabilitation were categorized and summarized in Figure 3. The majority of needs related to caring for their own diseases (62.78%), and the rest to promoting their own health and well-being (11.62%), such as learning how to maintain a healthier life, and clarifying what appropriate care is for the elderly. The most frequently requested home healthcare service pertained to musculoskeletal diseases (40.1%), and included requests for walking-aids, help recovering motor function, and relief from muscle-aches. Other needs for home healthcare services related to caring for those with cardiovascular disease (11.1%), and sensory/dermatological problems (7.6%).

## 4. Discussion

The results of this study are consistent with previous studies that found epidemiological patterns shifting from a predominance of communicable diseases to noncommunicable diseases in Vietnam [19, 20]. Of this study's participants, 79% self-reported having a chronic disease that increased their households' financial burdens. Interestingly, cardiovascular disease, diabetes, and cancer were rarely reported in this study, although they are the leading causes of death in Vietnam [21]. It is possible that elders may not be aware of the symptoms and progression of these diseases, and find them when they are in a later stage. Screenings and regular health checkups may be critical for the elderly population, to ensure early detection and disease prevention.

The study's findings are not consistent with those of a previous systematic literature review that found a significant relationship between recent smoking behaviors, alcohol consumption, and the level of functioning. We suspect that this is because the systematic literature review primarily included research studies that had been conducted in highly developed countries [21].

The results of this study were similar to those of Hoi et al.'s study [3], which reported that the HRQoL of older people in rural Vietnam was affected by economic status, educational level, and marital status. After controlling for the effects of socioeconomic variables, this study's regression analysis indicated that self-rated health status, BMIs, and the number of NCDs were significant predictors of HRQoL. Thus, the quality of life among older people may be greatly increased by moving toward healthy lifestyles that include healthy diets and exercise, chronic disease management, and subjective well-being.

Meanwhile, even though more people recognize the importance of healthy lifestyles in modern society, older people's smoking and drinking habits are not easily changed. An et al. [22] reported that adults living in urban areas were more knowledgeable about the harmful health effects of active and passive smoking than those living in rural areas, and these significant differences in knowledge were related to access to health information. The fact that more than half of the participants in this study required health information from health professionals indicates a need to develop strategies for providing information efficiently to elderly people in rural areas.

The results of this study documented the various needs for healthcare and rehabilitation services among Vietnam's elderly population. Physical disabilities and functional limitations are common among older people [23] and lead to adverse consequences such as dependency and morbidity, the increased utilization of healthcare, and a need for supportive services and long term care [24]. Hairi et al. [24] reported that the prevalence of functional limitations was 20% in older Malaysians, and advancing age, gender (more women than men), the presence of arthritis, and having a depressive symptomology were significantly associated with functional limitations. This study found that elders were in great need of obtaining health information to manage chronic illness and improve their health in general by adopting healthy lifestyles.

Along with the increase in the proportion of the population that is aging, and the increasing prevalence of non-communicable diseases, readily accessible community-based primary care is critical. A Vietnamese commune health center is located in a community which covers 25,000 people. The roles of these centers should be strengthened to address effectively the elderly population's needs for health care services, particularly in rural areas. The WHO has emphasized the importance of community-based primary health services. Community health centers may become critical healthcare service providers for managing chronic illnesses, promoting healthy lifestyles and behaviors, reducing health risks, preventing diseases, promoting health, and improving the elderly population's quality of life.

The study's findings are limited by certain factors. First, the study's participants were limited to the elderly living in the rural Quoc-Oai district of Hanoi, using stratified proportion sampling, and quota assignment. Thus, to validate the generalizability of the results for the rural elderly in Vietnam, the study should have involved more participants from different regions and districts. Second, the data was collected through a survey questionnaire, and the information obtained from the study participants was assumed to be accurate. Further research is required to examine the correlates and predictors of health outcomes, and to investigate the effectiveness and feasibility of community health center-based, nurse-led, chronic illness management programs for the rural elderly in Vietnam.

## 5. Conclusions

The aging proportion of Vietnam's population is growing rapidly. The burden of providing healthcare for non-communicable diseases continues to increase as a consequence. It is vital that health challenges and the healthcare needs of older adults be assessed. Furthermore, it is important that healthcare services be easily accessible and address the elderly population's increasing needs for chronic illness management, risk reduction, help adopting healthy lifestyles, and improving their quality of life.

## Figures and Tables

**Table 1:** Demographic characteristics of the elderly (*N* = 713).

Variables	*N*	%
Age	(Mean ± SD)	(70.9 ± 8.81)
60 s	377	52.9
70 s	194	27.2
Over 80	142	19.9

Gender		
Male	295	41.4
Female	418	58.6

Ethnicity		
Kinh	580	81.3
Non-Kinh	133	18.7

Location		
Plain	555	77.8
Mountain	158	22.2

Marital status		
Married/reunion	417	58.5
Single/divorced/separated	31	4.3
Widowed	265	37.2

Education		
Under primary	247	34.6
Primary	203	28.5
Secondary	182	25.5
High school and higher	81	11.4

Religion		
Yes	13	1.8
No	700	98.2

Working status		
Unemployed & retired	170	23.8
Still working	543	76.2

Household size	(Mean ± SD)	(4.64 ± 2.08)
1-2 people	152	21.3
3-4 people	148	20.8
5-6 people	290	40.7
Over 7 people	123	17.3

Wealth quintile		
Richest	134	18.8
Richer	131	18.4
Middle	157	22.0
Poorer	152	21.3
Poorest	139	19.5

Health insurance		
Yes	517	72.5
No	196	27.5

**Table 2:** Health status and chronic disease prevalence of sample (*N* = 713).

Variables	60 s		70 s		Over 80		Total	
	*n*	%	*n*	%	*n*	%	*n*	%
	Mean	SD	Mean	SD	Mean	SD	Mean	SD
Self-rated health status								
Good	89	23.6	20	10.3	11	7.7	120	16.8
Normal	212	56.2	115	59.3	70	49.3	398	55.7
Bad	76	20.2	59	30.4	61	43.0	196	27.5

BMI	20.6	2.57	19.4	2.63	18.9	2.78	19.9	2.72
Severely underweight	17	4.5	13	6.7	26	18.3	56	7.9
Moderately underweight	11	2.9	19	9.8	6	4.2	36	5.0
Mildly underweight	46	12.2	40	20.6	30	21.1	116	16.3
Normal	282	74.8	118	60.8	77	54.2	477	66.9
Overweight	21	5.6	4	2.1	3	2.1	28	3.9

Recent smoker^*∗*^ (*n* = 192)								
Yes	71	53.0	13	34.2	4	20.0	88	45.8

Drinks in a month^†^ (*n* = 198)								
Yes	111	79.3	22	64.7	12	50.0	145	73.2

Social capital								
Trust in daily life	4.17	0.497	4.17	0.465	4.19	0.507	4.17	0.490
Trust in urgent need	3.04	0.631	2.98	0.605	2.94	0.651	3.00	0.628
Total	3.54	0.496	3.51	0.478	3.49	0.473	3.52	0.487

Diagnosed chronic disease								
Hypertension	102	27.1	69	35.6	54	38.0	225	31.6
Diabetes	14	3.7	7	3.6	5	3.5	26	3.6
Cancer	1	0.3	2	1.0	1	0.7	4	0.6
Neuropsychiatric diseases	16	4.2	5	2.6	6	4.2	27	3.8
Cardiovascular diseases	32	8.5	14	7.2	10	7.0	56	7.9
Respiratory diseases	20	5.3	11	5.7	14	9.9	45	6.3
Asthma/COPD	19	5.0	10	5.2	9	6.3	38	5.3
Liver-related diseases	23	6.1	5	2.6	1	0.7	29	4.1
Kidney-related diseases	29	7.7	10	5.2	5	3.5	44	6.2
Gastrointestinal diseases	82	21.8	28	14.4	13	9.2	123	17.3
Musculoskeletal diseases	180	47.7	103	53.1	79	55.6	362	50.8
Movement impaired	11	2.9	3	1.5	4	2.8	18	2.5
Others	46	12.2	31	16.0	19	13.4	96	13.5

^*∗*^The person who smokes at the present. ^†^The person who has used alcohol beverages in the last month.

**Table 3:** Functioning assessment according to general characteristics.

Variables	Functional limitation		*p*	*Post hoc* ^‡^
	Mean	SD		

Age groups				
60 s^a^	1.609	0.811	0.000	a < b < c
70 s^b^	2.012	0.940		
Over 80^c^	2.548	1.089		

Gender				
Male	1.718	0.887	0.000	
Female	2.038	1.015		

Ethnicity				
Kinh	1.936	0.981	0.086	
Non-Kinh	1.775	0.947		

Location				
Plain	1.940	0.987	0.080	
Mountain	1.786	0.932		

Marital status				
Married/reunion^a^	1.766	0.942	0.000	a < c
Single/divorced/separated^b^	1.906	0.904		
Widowed^c^	2.126	1.001		

Education				
Under primary^a^	2.202	1.101	0.000	a > b, c, d
Primary^b^	1.850	0.909		
Secondary^c^	1.667	0.817		
High school and higher^d^	1.681	0.838		

Religion				
Yes	1.730	0.627	0.514	
No	1.909	0.982		

Working status				
Unemployed & retired	2.061	1.063	0.017	
Still working	1.857	0.944		

Household size				
1-2 people	1.875	0.929	0.766	
3-4 people	1.848	0.946		
5-6 people	1.942	0.979		
Over 7 people	1.930	1.067		

Wealth quintile				
Richest	1.800	0.880	0.083	
Richer	1.874	0.910		
Middle	1.840	0.959		
Poorer	2.096	1.073		
Poorest	1.905	1.018		

Health insurance				
Yes	1.940	0.997	0.134	
No	1.817	0.917		

Recent smoker^*∗*^				
Yes	1.572	0.786	0.055	
No	1.828	1.012		

Drinks in a month^†^				
Yes	1.574	0.759	0.000	
No	2.142	1.184		

Self-rated health status				
Good^a^	1.310	0.592	0.000	a < b < c
Normal^b^	1.739	0.806		
Bad^c^	2.608	1.086		

BMI				
Severely underweight^a^	2.591	1.214	0.000	b, c, d, e < a d < c
Moderately underweight^b^	2.047	0.921		
Mildly underweight^c^	2.004	0.971		
Normal range^d^	1.769	0.924		
Overweight^e^	1.814	0.851		

Number of NCDs				
0^a^	1.505	0.625	0.000	a < b, c < d
1^b^	1.894	0.846		
2^c^	2.016	0.913		
3 and over^d^	2.233	0.964		

^*∗*^ *n* = 192, ^†^*n* = 198; ^‡^LSD.

**Table 4:** Health-related quality of life according to socioeconomics and health status.

Variables	Health-related quality of life		*p*	*Post hoc* ^‡^
	Mean	SD		

Age groups				
60 s^a^	0.883	0.140	0.000	a > b > c
70 s^b^	0.838	0.148		
Over 80^c^	0.759	0.239		

Gender				
Male	0.872	0.140	0.001	
Female	0.828	0.190		

Ethnicity				
Kinh	0.840	0.172	0.048	
Non-Kinh	0.873	0.172		

Location				
Plain	0.840	0.171	0.059	
Mountain	0.869	0.175		

Marital status				
Married/reunion^a^	0.862	0.160	0.013	a > c
Single/divorced/separated^b^	0.833	0.214		
Widowed^c^	0.823	0.184		

Education				
Under primary	0.797	0.216	0.000	a < b, c, d
Primary	0.858	0.858		
Secondary	0.886	0.124		
High school and higher	0.879	0.123		

Religion				
Yes	0.847	0.110	0.980	
No	0.846	0.173		

Working status				
Unemployed & retired	0.826	0.196	0.076	
Still working	0.853	0.164		

Household size				
1-2 people	0.860	0.138	0.145	
3-4 people	0.865	0.117		
5-6 people	0.839	0.192		
Over 7 people	0.824	0.211		

Wealth quintile				
Richest	0.860	0.142	0.358	
Richer	0.845	0.176		
Middle	0.862	0.163		
Poorer	0.827	0.160		
Poorest	0.837	0.215		

Health insurance				
Yes	0.842	0.179	0.288	
No	0.857	0.155		

Recent smoker^*∗*^				
Yes	0.880	0.141	0.416	
No	0.863	0.137		

Drinks in a month^†^				
Yes	0.889	0.119	0.593	
No	0.877	0.175		

Self-rated health status				
Good^a^	0.932	0.048	0.000	a > b > c
Normal^b^	0.878	0.117		
Bad^c^	0.730	0.244		

BMI				
Severely underweight^a^	0.739	0.234	0.000	a < b, c < d
Moderately underweight^b^	0.789	0.217		
Mildly underweight^c^	0.825	0.180		
Normal range^d^	0.867	0.154		
Overweight^e^	0.872	0.123		

Number of NCDs				
0^a^	0.914	0.072	0.000	a > b, c > d
1^b^	0.854	0.167		
2^c^	0.832	0.177		
3 over^d^	0.772	0.221		

^*∗*^ *n* = 192, ^†^*n* = 198; ^‡^LSD.

**Table 5:** Multiple regression coefficients for significant factors of HRQoL.

Variables^†^	Model 1			Model 2		
	*β* ^*∗*^	SE	*p*	*β*	SE	*p*

Constant	0.886	0.040		1.006	0.047	
Age group	−0.051	0.009	0.000	−0.032	0.008	0.000
Gender	−0.024	0.014	0.096	−0.016	0.013	0.226
Ethnicity	0.037	0.016	0.017	0.019	0.015	0.203
Marital status	0.004	0.008	0.608	0.002	0.007	0.833
Education	0.015	0.007	0.036	0.010	0.007	0.142

Self-rated health				−0.076	0.010	0.000
BMI				0.018	0.006	0.005
Number of NCDs				−0.022	0.006	0.000

*R* ^2^	0.093			0.233		

^*∗*^Unstandardized coefficients; ^†^reference category: age 60–69, male, non-Kinh, married/reunion, under primary school, good self-rated health, severely underweight, and having no NCDs.
