# Corrigendum to “Health Status and the Demand for Healthcare among the Elderly in the Rural Quoc-Oai District of Hanoi in Vietnam”

**DOI:** 10.1155/2019/9590417

**Published:** 2019-04-04

**Authors:** Kyung-Sook Bang, Sunghee H. Tak, Juhwan Oh, Jinseon Yi, Soo-Young Yu, Truong Quang Trung

**Affiliations:** ^1^The Research Institute of Nursing Science, College of Nursing, Seoul National University, Seoul, Republic of Korea; ^2^JW Lee Center for Global Medicine, College of Medicine, Seoul National University, Seoul, Republic of Korea; ^3^College of Nursing, Seoul National University, Seoul, Republic of Korea; ^4^Faculty of Nursing and Midwifery, Hanoi Medical University, Hanoi, Vietnam

In the article titled “Health Status and the Demand for Healthcare among the Elderly in the Rural Quoc-Oai District of Hanoi in Vietnam” [1], additional information should be added to the Acknowledgments section and it should be updated as follows:

“This research was financially supported by the JW LEE Center for Global Medicine of Seoul National University College of Medicine, Seoul, Republic of Korea. This project is part of a collaborative project by Hanoi University of Public Health, Hanoi Medical University, University of Medicine and Pharmacy of Ho Chi Minh City, and the JW LEE Center for Global Medicine of Seoul National University College of Medicine, Seoul, South Korea.”
